# Nurses’ perception of talent management scale (NPTMS): development, validation and psychometric properties

**DOI:** 10.1186/s12912-025-02882-8

**Published:** 2025-04-03

**Authors:** Duygu Gül, Betül Sönmez

**Affiliations:** 1 Department of Nursing, Faculty of Health Sciences, Çanakkale Onsekiz Mart University, Çanakkale, Türkiye; 2 Barbaros Neighborhood, Çanakkale Onsekiz Mart University Terzioğlu Campus, Prof. Dr. Sevim Buluç Street, No:20, Çanakkale, Sarıcaeli 17100 Türkiye; 3 Department of Nursing Management, Florence Nightingale Faculty of Nursing, Istanbul University-Cerrahpaşa, Istanbul, Türkiye; 4 Florence Nightingale Faculty of Nursing, Istanbul University-Cerrahpaşa, Abide-i Hürriyet Street, Istanbul, Şişli 34381 Türkiye

**Keywords:** Instrument development, Nursing, Psychometric properties, Scale development, Talent management, Talent management in nursing, Validity, Reliability

## Abstract

**Background:**

There is an increasing need for a new and comprehensive approach to evaluate nursing talent to increase effectiveness and productivity. Talent management, which plays an important role in identifying, developing and retaining nursing talent, is a key strategy for investing in nursing. This study aimed to develop the Nurses’ Perception of Talent Management Scale (NPTMS) and assess its psychometric properties.

**Methods:**

The scale was developed using a methodological design with a convenience sampling method including 918 nurses (n_EFA_=422, n_CFA_=496) from 12 hospitals in Istanbul between September and April 2022. The scale was developed in three phases. Firstly, items reflecting talent management in nursing were created through a comprehensive literature review employing the deductive method. Then, the face and content validity of the scale were evaluated. Finally, construct validity (exploratory and confirmatory factor analysis, concurrent validity, convergent and divergent validity) and reliability (item-total score correlation, split-half method, Cronbach’s α coefficient, equivalent forms reliability and test-retest) were evaluated for psychometric properties.

**Results:**

The newly developed scale, for which validity and reliability analyses were conducted using two separate samples through exploratory and confirmatory factor analysis, was found to consist of 26 items and a single factor. This factor explained 63.2% of the variance related to the structure and showed acceptable goodness of fit (χ2/sd = 4.325, RMSEA = 0.078, RMR = 0.046, TLI = 0.915, CFI = 0.924, NFI = 0.903, GFI = 0.882, IFI = 0.924). The content validity of the scale was found to be 0.95. Construct validity results indicated that the scale exhibited strong concurrent validity (r_EFA_ =0.755, r_CFA_ =0.772, *p* < 0.05) and convergent and divergent validity (AVE > 0.5; CR > 0.8; CR > AVE). The reliability analyses revealed high internal consistency (0.976_EFA_;0.978_CFA_), time invariance (ICC = 0.836), and equivalent forms reliability (*p* < 0.05).

**Conclusions:**

The scale is a valid and reliable tool for assessing nurses’ perceptions of talent management. It can be used to evaluate talent management practices in nursing and developing policies and strategies that support investment in nursing talent.

**Clinical trial number:**

Not applicable.

**Supplementary Information:**

The online version contains supplementary material available at 10.1186/s12912-025-02882-8.

## Introduction

The nursing shortage presents global challenges for healthcare institutions [1, 2]. The World Health Organization (WHO) highlights the importance of attracting, deploying and retaining nurses to increase the efficiency and productivity of the workforce, as nurses represent the majority of the health workforce [3]. Similarly, the International Council of Nurses (ICN) has emphasised the need to recognize the skills, qualifications, and abilities of nurses, noting that investing in nursing provides economic and social benefits [4]. The COVID-19 pandemic, which caused significant changes in the provision of healthcare services, also highlighted the necessity of investing in nurses by demonstrating the critical need for talented nurses to adopt to rapidly evolving healthcare environments [5]. A study shows that the quality of nursing care provided by talented nurses is higher [6].

Although there is no universally agreed-upon definition of talent, it is generally described as the systematic development or mastery of skills [7], high potential [8, 9], excellent performance [8, 10], unique, rare and inimitable core competencies [10] and strategic value [9]. In a study by Haines [11], nursing talent is defined as the ability of nurses to use their leadership qualities through professional knowledge and skills. This study further characterizes a talented nurse as someone who can anticipate patient needs, recognize and empower patients as individuals, advocate for patients, serve as a role model, and provide exceptional care. In this context, it is suggested that “talent management (TM)” can be used as an effective strategy for managing the talented nurse workforce [5].

According to the Resource-Based View, which provides a theoretical foundation for the importance of talent management, an organization’s valuable, rare, inimitable, and irreplaceable resources and capabilities are crucial for achieving high performance and gaining a competitive advantage [12]. The Human Resource Architecture Approach, which emphasizes the strategic value and uniqueness of human resources [13], argues that a single, standardized human resources architecture is unsuitable for managing employees across all organizations. The Talent Factory Model (2008), which frames every organization as a talent factory, provides a scientific framework for recruiting, developing, placing, and retaining talent, outlining how to establish such a talent factory [14]. Bersin’s New Talent Management Framework (2010) focuses on practices related to attracting, developing, managing, and retaining key employees within the organization [15]. Finally, the Classical Model: Systems Approach [16] highlights the importance of coordinating these practices for effective implementation.

In the literature, TM in nursing is defined as a systematic process of various practices such as identifying, attracting, recruiting, placing, developing and retaining talent [17]. *Talent identification* involves recognizing the talents and qualities that are currently needed and will be required in the future (e.g., leadership potential, performance and potantial, career desire, adaptability and willingness to learn) [18, 19]. Measurement criteria are established by assessing performance, potential and competencies with results placed into a talent matrix, which categorizes the workforce into different segments [20]. *Talent attraction* refers to the ability to draw talented employees to the organization and communicate the right message to the right individuals [20]. In this phase, it is aimed to identify and attract innovative, creative, high-potential, and high-performance employees [21]. Previous studies suggest that magnet hospitals [22], which provide nurses with opportunities for both horizontal and vertical career advancement, have clear development policies [23] and are effective in attracting nurses. During the pandemic, the United States of America (USA) addressed the shortage of qualified nurses by employing experienced travel nurses and providing a range of incentives, including salary adjustments, housing support, bonuses, and social assistance [24]. *Talent recruitment* involves evaluating the talents that an organization may need though a continuous, talent-focused approach, in contrast to traditional recruitment procedures [20, 21]. A study found that nurse managers prioritize personal characteristics, educational level, experience and competence when recruiting nurses [25]. *Talent placement* ensures that employees are assigned to position where they can effectively use their talents [18]. In a study conducted during the pandemic [26] selection and placement criteria were established qualified nurses to work in COVID-19 units. *Talent development*, on the other hand, focuses on enhancing employees’ attitudes and skills [27]. At this stage, talent development practices focusing on an individual’s performance, potential and areas for improvement, and specific talents, vary depending on whether talent is treated as inclusive or exclusive [9, 18, 20]. The inclusive approach, which assumes that all employees have talent [8, 19], applies talent development practices to the entire workforce. In contrast, the exclusive approach, which suggests that only a small number of individuals are truly talented [9, 19], develops the talents identified through performance and potential assessments based on the future needs of key positions and individual development requirements (i.e., talent pool) [14, 20]. However, a study suggests that there is no talent pool in nursing [28]. The development of talent in nursing is supported through a variety of strategies, including training and coaching, orientation programs, electronic learning, leadership development and career advancement initiatives [20]. In addition, individualized projects, online training platforms, emergency task management skills, and empowerment through delegation [18] are essential components of effective talent development in addition to motivation and continuous education [29]. *Talent retention*, on the other hand, refers to the strategies and practices that ensure talented employees, who contribute significantly to the organization, remain engaged and committed for the long term [20]. In previous studies, several key factors in retaining nursing talent, including talent management program [1], continuous professional education, career development opportunities, additional payments [30], and the implementation of magnet hospital standards [22], are reported to contribute to enhancing nurse retention.

Studies on TM in nursing indicates that it provides mutual benefits for nurses, healthcare services and more holistic health systems [1, 6, 27, 30–34]. However, existing studies on TM in nursing remain relatively limited [1]. It is noted that TM is not considered as a comprehensive, organizational process in nursing; and therefore, TM practices are often addressed separately [11]. TM practices such as recruitment, leadership development, succession planning and retention are used in the nursing literature [35]. However, despite considerable attention to practices such as retention and development, the complete scope and content of TM in nursing remains unexplored. There is a critical need for further theoretical exploration of TM practices in nursing, in addition to correct and effective application [1].

The study focused on addressing the theoretical ambiguity regarding TM in nursing, the uncertainties regarding its processes, and the lack of a valid and reliable measurement tool for assessing TM practices in this context. While various TM scales and questionnaires have been developed in both Turkish and English for employees in different sectors, these tools are insufficient for measuring nurses’ perceptions of TM and evaluating TM practices within nursing services. Many of the elements assessed by these instruments, developed for other disciplines, may not be relevant or applicable to the nursing context. The nursing profession faces distinct challenges, including demographic shifts, technological advancements, workforce changes, and the need for cost-effective practices. In addition, the profession contends with the pressures of attracting and retaining a talented workforce capable of adapting to the increasingly complex and rapidly evolving healthcare environment, influenced by factors such as competition and globalization. Given the unique characteristics of nursing practice environments, it is crucial to develop a valid and reliable measurement tool specifically designed to evaluate TM practices within nursing as a holistic organizational process. The assessments of nurses regarding talent management within their institutions will contribute to identifying the strengths and weaknesses of current nursing service delivery, as well as revealing the gap between nurses’ expectations and the practices of nurse managers/leaders in this regard. Furthermore, the development of talent management practices will promote the design of nursing service delivery from a talent-focused perspective. The development and evaluation of nurses’ talents may lead to positive outcomes for nurses, patients, and the organization. In this context, the aim of this study was to develop the Nurses’ Perception of Talent Management Scale (NPTMS) and to determine its psychometric properties for assessing nurses’ perceptions of talent management.

## Methods

### Study design

This study employed a methodological design.

### Study procedure

The study was conducted in three phases: the development of the NPTMS, evaluation of face and content validity, and, finally, assessment of construct validity and reliability to determine the psychometric properties.

### Phase 1: scale development

#### Conceptualization

**Fig. 1:**
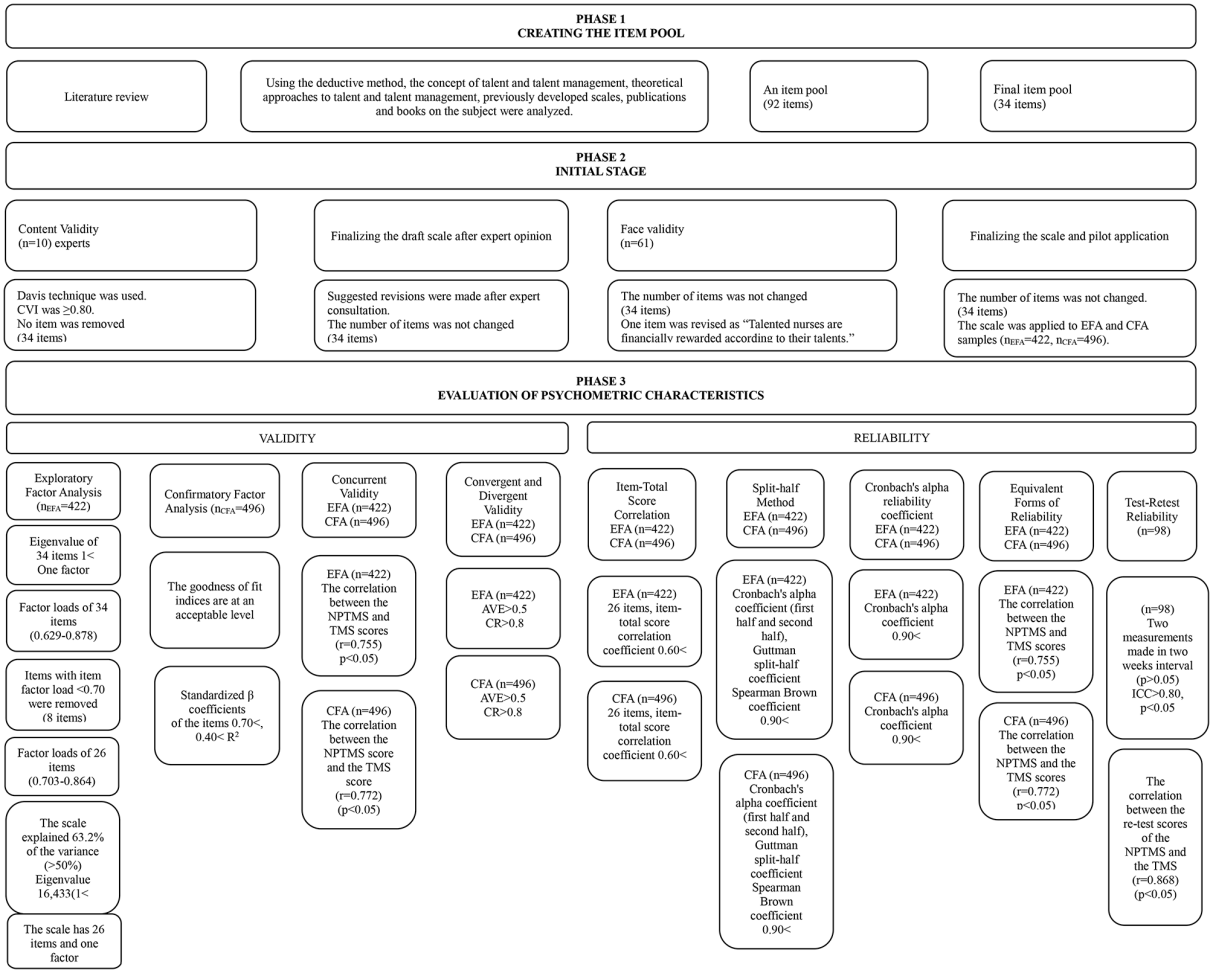
Stages of development of the scale

In accordance with the fundamental principles of scale development, the NPTMS was developed in three phases [36–38] (Fig. 1). Initially, a comprehensive literature review was conducted using a deductive approach, and an item pool was created based on existing scales and sources [36]. Theoretical frameworks related to TM were examined, including Social Change Theory (SET) [39], the Resource-Based View [12], Core Competence Theory [40], Mckinsey Research [41], the Human Resources Architecture Approach [13], the Talent Factory Model [14], the Talent Farm Model [42], the Classical Model in Talent Management: Systems Approach [16] and Bersin’s New Talent Management Framework [15]. These theories provided the foundation for the theoretical structure of TM. In addition, TM scales developed in both Turkish (10) (e.g [43]). and English (4) (e.g [10]) as well as relevant questionnaires (2), such as *the Job Crafting Scale* [44] (1), *the Talent Development and Leadership Development Practices Index* (1) [45], and books on TM (e.g [18, 20, 21]) were also reviewed to inform the evaluation of TM.

It is necessary to consider all relevant factors of the construct to be measured when generating scale items [36, 37]. In the development of the NPTMS, a through examination conducted in addition to the previously cited literature (e.g [9, 21, 27]). Based on the findings, the structure of the NPTMS was established, incorporating items that reflect the key components of the TM process, including talent identification, attraction, recruitment, placement, development and retention practices.

#### Item generation

The scale items were formulated with careful attention to ensuring that each item contained a clear judgement, was comprehensible, and aligned with the structure of the NPTMS as defined during the conceptualization process [38]. Initially, the item pool consisted of 92 items. This pool was then reduced to 34 items based on criteria such as content relevance, redundancy, adherence to grammatical rules, and overall clarity.

### Phase 2: content validity and face validity

#### Content validity

Content validity was assessed using the Davis technique with a 4-point Likert scale, based on the evaluations of 10 experts [46]. In this study, the Content Validity Index (CVI) values for the 34 items ranged from 0.80 to 1.00, with an overall CVI of 0.95 for the scale, confirming its content validity [46, 47]. At this stage, the number of items remained unchanged (34 items), and the items adequately represented the intended construct.

#### Face validity

To assess the intelligibility and linguistic characteristics of the scale, it was applied to a sample of 61 nurses [37], including nurse managers and nurses with similar characteristics to the target group. These nurses, who were employed at different hospitals and had varying educational backgrounds, were not included in the final sample. At this stage, one item was revised to read “Talented nurses are financially rewarded according to their talents”. The average duration allocated by to complete the scale was 10–15 min., and they reported no confusion or difficulty in understanding the items, indicating that the scale was clear and straightforward. The language and spelling were thoroughly reviewed, and necessary adjustments were made. No items were removed, and the overall the suitability and legibility of the scale were confirmed.

### Phase 3: psychometric evaluation

In the psychometric evaluation of the scale, both construct validity (exploratory and confirmatory factor analysis, concurrent validity, convergent and divergent validity) and reliability (item-total score correlation, split-half method, Cronbach’s α coefficient, equivalent forms reliability and test-retest) were assessed.

#### Samples and data collection

The study population consisted of nurses working across 12 hospitals located in a metropolitan province (one public hospital, one training and research hospital, six private/foundation hospitals, four private/foundation university hospitals) (N_total_=2050). To ensure representativeness, hospitals were selected based on their ownership status (public, university and private/foundation hospitals) and their professional human resource management practices. Hospitals were identified through a non-probability sampling method, and data collection was performed in institutions that granted written permission. In scale development studies, it is recommended to include at least 10 participants (common) per item [36, 48], with 15 (ideal) [48] or 20 (high) for generalizability [37, 48]. Given that the scale in this study consisted of 34 items, the target sample size was a minimum of 340 nurses (34 × 10). Accordingly, the study was conducted using a convenience sampling method with 918 nurses (n_EFA_=422, n_CFA_=496) who met the inclusion criteria: having completed a two-month trial and orientation period, actively working during data collection and volunteering to participate. Data collection was conducted between September and April 2022 with a response rate of 44.7%.

### Talent management scale (TMS)

For the assessment of concurrent validity and equivalent forms reliability of the NPTMS, the TMS, developed by Tutar et al. (43), was applied concurrently with the NPTMS. The TMS, which consists of 18 items and a single factor, uses a 5-point Likert scale (1 = Never, 5 = Always), where higher scores indicate a stronger perception of talent management practices. The Cronbach’s α coefficient of the original TMS was reported as 0.93 (43), whereas in this study, it was 0.975.

### Ethical considerations

Prior to conduct of the research, approval was obtained from the Ethics Committee of Istanbul University-Cerrahpaşa (Date: 08.06.2021; Number: 107065), as well as from the hospital administrations and the Provincial Health Directorate. The study was conducted in accordance with the principles outlined in the Declaration of Helsinki. Approval for the use of the TMS was received via email from the author who developed the scale. Prior to the application of data collection tools, nurses were informed about the research (purpose, duration, voluntary participation, confidentiality, access to the researcher, the right to withdraw at any time, etc.). During the data collection process, each participant was provided with an Informed Consent Form and the data collection tools in a sealed envelope. Nurses signed the consent form, and after completing the data collection tools, they returned both documents in the same sealed envelope to the researcher. The returned forms were securely stored in a locked cabinet, and the data were maintained on an encrypted computer. Throughout the data analysis process, confidentiality was ensured by the statistical consultant.

### Data analysis

Data analysis was conducted using SPSS 24.0 (IBM^®^ SPSS^®^ Corp, Armonk, New York) and AMOS GRAPHICS 21. A total of 36 forms, where a significant portion of the scales were incomplete, were excluded from the analysis. The missing data ranged from 0.02 to 0.09%, and median values were assigned to the missing data.

Exploratory Factor Analysis (EFA) (n_CFA_=422) was conducted to assess the construct validity of the NPTMS, and Confirmatory Factor Analysis (CFA) was applied to a different sample than EFA (n_CFA_=496) [37, 49] to validate the obtained structure. The personal and professional characteristics of the nurses in the EFA and CFA samples were compared using the $\:{\chi\:}^{2}$ test. The suitability of the data for factor analysis was assessed using the Kaiser-Meyer-Olkin (KMO) and Bartlett’s test of sphericity. The Principal Component Analysis (PCA) method was used for factor extraction, and the number of factors was determined using the eigenvalue method, the scree plot and the explained variance ratios [36–38]. Before conducting the EFA and CFA, Mahalanobis distance was evaluated through extreme value analysis. Based on the range of standardized z scores (-3, + 3), three data in the EFA sample and nine data in the CFA sample were identified as outliers and excluded from the analysis [50]. For concurrent validity, the TMS was applied to both the EFA and CFA samples, and the Pearson correlation coefficient [51] was calculated. The Average Variation Extracted (AVE) and Composite Reliability (CR) values were calculated for convergent and divergent validity of the EFA and CFA samples [36, 52]. In the CFA, the goodness of fit indices were used to evaluate the model fit, and item significance was assessed using the standardized β coefficient.

To determine the reliability of the NPTMS, several methods were employed following both EFA and CFA. These included item-total score correlation, the split-half method, the Guttman split-half and Spearman-Brown coefficients, Cronbach’s α, equivalent forms reliability and test-retest reliability. The test-retest reliability was assessed using the CFA sample, with analyses conducted on at least 25% of the sample (*n* = 98 nurses) at two-weeks intervals. Paired sample t-tests and intraclass correlation coefficients (ICC) were calculated to assess stability over time. Normality analyses for both the NPTMS and the TMS indicated that the skewness and kurtosis coefficients were within the acceptable range for a normal distribution (-1, + 1) [53]. Descriptive statistics, including mean, standard deviation, minimum, maximum, mode, and median, were used. The differences between institutions were analyzed using an ANOVA test. All statistical analyses were conducted at a significance level of 5%.

## Results

### Personal and professional characteristics of nurses for the EFA and CFA samples

No significant differences were found between the EFA and CFA samples with regard to age (χ2 = 4.856), gender (χ2 = 0.795), marital status (χ2 = 0.153), duration of institutional experience (χ2 = 1.877), and duration of professional experience (χ2 = 2.995) (*p* > 0.05), indicating a homogeneous distribution (Table 1).

**Table 1:** Personal and professional characteristics of nurses for exploratory factor analysis and confirmatory factor analysis samples (N_EFA_=422, N_CFA_=496)

Variable	Subgroup	EFA		CFA		Total			
		*n*	%	*n*	%	*n*	%	χ2	*p*
Age	≤ 25 26–30 31 ≤	228 97 97	54.0 23.0 23.0	232 135 129	46.8 27.2 26.0	460 232 226	50.1 25.3 24.6	4.856	0.088
Gender	Female Male	346 76	82.0 18.0	410 86	82.7 17.3	756 162	82.4 17.6	0.795	0.428
Educational Status	Health vocational school Associate degree Bachelor’s Master’s	126 77 194 25	29.9 18.2 46.0 5.9	88 96 248 64	17.7 19.4 50.0 12.9	214 173 442 89	23.3 18.8 48.1 9.7	26.730	*0.000
Marital Status	Married Single	122 300	28.9 71.1	166 330	33.5 66.5	288 630	31.4 68.6	0.153	0.079
Institution	Private hospital/ Foundation hospital	156	37.0	205	41.3	361	39.9	1.877	0.598
	Private university hospital/ Foundation university hospital	134	31.8	147	29.6	281	30.6		
	Training and research hospital	89	21.1	99	20.0	188	20.5		
	Public hospital	43	10.2	45	9.1	88	9.6		
Position	Nurse manager Special nurse Nurse	53 30 339	12.6 7.1 80.3	74 87 335	14.9 17.5 67.5	127 117 674	13.8 12.7 73.5	25.747	*0.000
Unit	Surgical Internal Emergency Operating room Administrative units Intensive care Polyclinic Mixed	73 156 18 42 8 76 17 32	17.3 37.0 4.3 10.0 1.9 18.0 4.0 7.6	61 231 25 18 35 72 20 34	12.3 46.6 5.0 3.6 7.0 14.5 4.0 6.9	134 387 43 60 43 148 37 66	14.6 42.2 4.7 6.5 8.9 16.1 4.0 7.2	38.246	*0.000
Duration of Institution Experience	< 1 year 1–5 years 6–10 years 11 years ≤	157 158 61 46	37.2 37.4 14.5 10.9	177 152 94 73	35.7 30.6 19.0 14.7	334 310 155 119	36.4 33.8 16.9 13.0	8.556	*0.044
Duration of Professional Experience	< 1 year 1–5 years 6–10 years 11 years ≤	43 210 83 86	10.2 49.8 19.7 20.4	64 222 96 114	12.9 44.8 19.4 23.0	107 432 179 200	11,5 47.2 19.5 21.8	2.995	0.392
Working Schedule	Day Shift	130 292	30.8 69.2	210 286	42.3 57.7	340 578	37.0 63.0	13.005	*0.000
Certificate Status	Yes No	214 208	50,7 49.3	285 211	57.5 42.5	499 419	54.4 45.6	4.186	*0.046
Compliance of the Certificate to the Unit	Yes No	152 62	71.0 29.9	223 62	78.2 21.8	375 124	71.1 24.9		

Note. n: Frequency, %: Percentage, χ2 Chi-Square Test, *p < 0.05, EFA: Exploratory Factor Analysis, CFA: Confirmatory Factor Analysis, of nurses in EFA who held a master’s degree branch of 17 was Nursing, of 6 was Health Institutions Management, of 2 was Other. At CFA, 44 of the nurses held a master’s degree in Nursing and 20 in Health Institutions Management

### Construct validity

EFA (n_EFA_=422) and CFA (n_CFA_=496) were conducted on separate samples to assess construct validity [37, 49].

### Exploratory factor analysis (EFA) sample

**Fig. 2:**
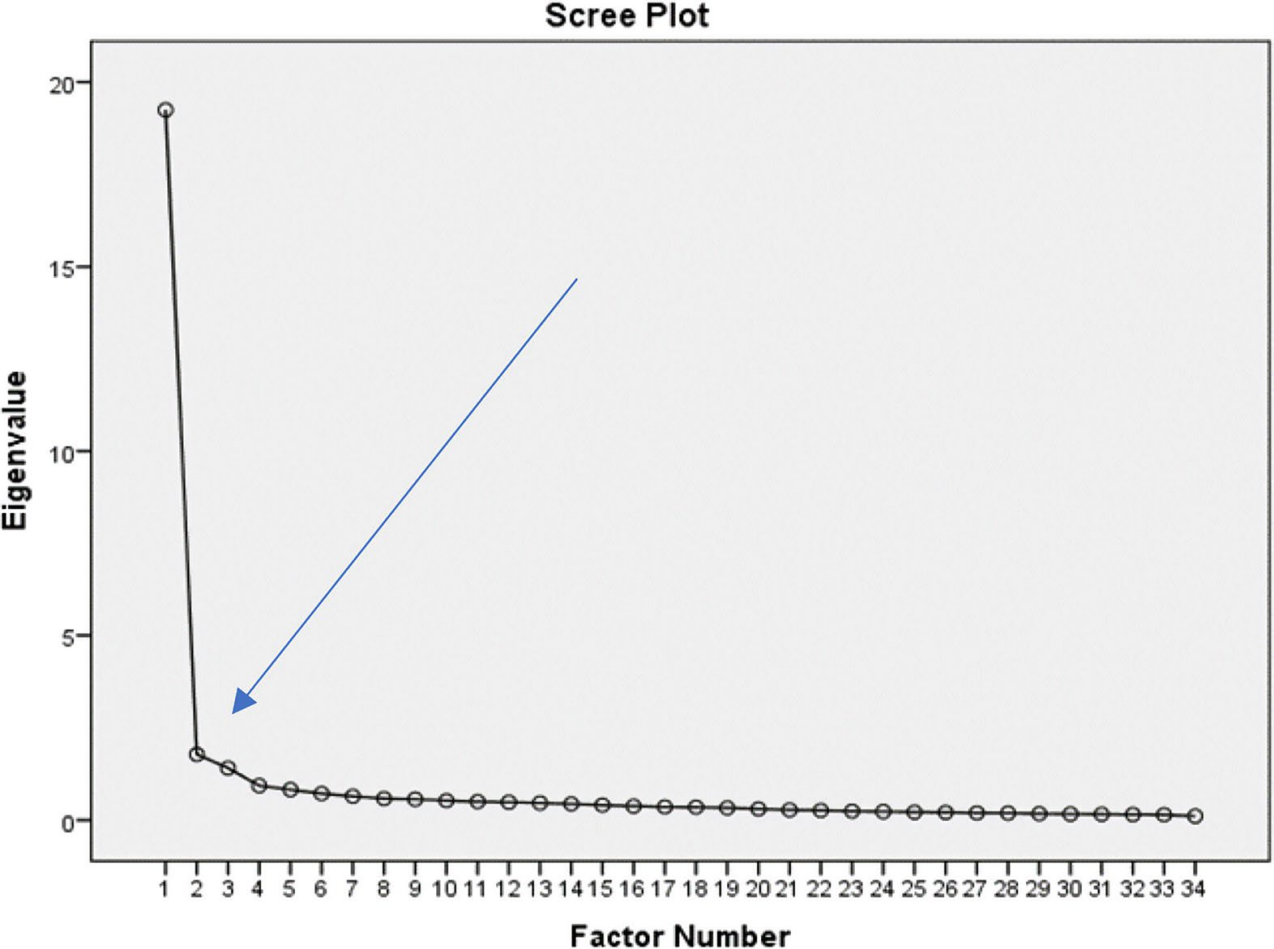
Scree plot graph of exploratory factor analysis

The EFA was conducted on a sample of 422 nurses (n_EFA_=422), approximately 12 times the number of items in the scale. The KMO coefficient was 0.972, indicating an excellent level of sampling adequacy (> 0.90) (50). Bartlett’s test of sphericity yielded χ² = 11.972.774, *p* = 0.000, *p* < 0.001 [54], confirming the suitability of the sample size for factor analysis and demonstrating a high correlation among the variables [50, 55]. Using the PCA, the EFA revealed a single-factor structure that explained 57.957% (> 50%) of the total variance, with an eigenvalue greater than 1 for the 34-item scale (Fig. 2) [37]. The factor loadings ranged from 0.629 to 0.878 (> 0.60), indicating high factor strength [56]. Loadings above 0.70 were considered significant, further supporting a well-defined structure [57]. Since the scale was unidimensional, the threshold for factor loading was set at 0.70 [58]. Accordingly, 8 items with factor loadings below 0.70 (items 1, 2, 3, 4, 6, 14, 17, and 28) were removed. After this adjustment, the factor loadings for the remaining 26 items ranged from 0.703 to 0.864, reflecting high factor strength [56] and a well-defined structure [57]. As a result, the refined scale explained 63.205% of the variance in the perceived TM structure, with an eigenvalue of 16.433 (> 1) (Table 2).

**Table 2:** The validity and reliability findings of the exploratory factor analysis sample (N_EFA_=422)

Item No	Factor Loads	M	SD	Item Total *r*	α when item is removed	α	AVE	CR	
NPTMS5- Nurses’ talent is determined using valid methods.	0.703	3.2339	1.04747	0.690	0.976				
NPTMS7- Talented nurses are provided with a work environment where they can demonstrate their talents.	0.764	3.2185	1.06784	0.750	0.976				
NPTMS8- Talented nurses are adequately compensated for their contribution to the organization.	0.739	2.7249	1.15942	0.716	0.976				
NPTMS9- Nurses are grouped according to their competencies and potential talents.	0.783	2.9769	1.08503	0.772	0.976				
NPTMS10- Talent management is an organizational policy.	0.720	3.2005	1.10803	0.707	0.976				
NPTMS11- Talented nurses are made to feel valued.	0.746	2.8406	1.16446	0.742	0.976				
NPTMS12- Training and development programs are organized according to the needs of the talented nurse.	0.745	3.0694	1.08780	0.746	0.976				
NPTMS13- The organization is known for its talented nurses.	0.739	3.0848	1.09380	0.704	0.976				
NPTMS15-Talented nurses with high adaptability to changing conditions are selected.	0.775	3.0617	1.11978	0.764	0.976				
NPTMS16- Nurses are given the opportunity to use their talents in their work.	0.806	3.2391	1.08263	0.789	0.975				
NPTMS18- Talented nurses are discovered through events for students and new graduates.	0.767	2.9846	1.14638	0.747	0.976				
NPTMS19- Nurses are given different roles and responsibilities in which they can develop their talents.	0.787	3.1594	1.09134	0.786	0.975				
NPTMS20- In recruitment, importance is given to matching the values of the organization with the individual values of talented nurses.	0.762	3.1388	1.05579	0.761	0.976	0.976	0.619	0.987	
NPTMS21- Managers are aware of nurses’ talents.	0.758	3.2159	1.07916	0.744	0.976				
NPTMS22- Talented nurses are tried to be recruited to the organization.	0.830	3.1774	1.13378	0.819	0.975				
NPTMS23- Nurses’ talents are compatible with the position they work in.	0.793	3.1825	1.06495	0.789	0.975				
NPTMS24- Nurses are developed in a talent pool for future positions.	0.806	2.9769	1.12926	0.793	0.975				
NPTMS25- The talents needed for each nursing position are determined.	0.847	3.1362	1.06947	0.835	0.975				
NPTMS26- Emphasis is placed on the talents of nurses when assigning them in key positions.	0.792	3.1877	1.08801	0.785	0.975				
NPTMS27- There are development opportunities for career advancement for talented nurses.	0.806	3.0951	1.08109	0.794	0.975				
NPTMS29- Nurses who feel that they cannot use their talents sufficiently in their work are given the opportunity to work in a position that matches their talents.	0.785	3.0540	1.08631	0.782	0.975				
NPTMS30- The most talented nurses are selected to provide added value to the organization.	0.823	2.9460	1.10746	0.815	0.975				
NPTMS31- The achievements of talented nurses are recognized.	0.791	3.0308	1.16834	0.776	0.976				
NPTMS32- Nurses are assigned in areas/units suitable to their talents.	0.854	3.1208	1.09040	0.838	0.975				
NPTMS33- Nurses are given the opportunity to use different methods in their work to utilise their talents more effectively.	0.864	3.0668	1.05060	0.863	0.975				
NPTMS34- Managers contribute to the development of nurses’ talents.	0.863	3.1568	1.10229	0.844	0.975				
Eigenvalue									16.433
Percentage of the Variation									63.205%

Note. NPTMS: Nurses’ Perception of Talent Management Scale, M: Mean, SD: Standard Deviation, r: Correlation, α: Cronbach’s alpha, AVE: Average Variance Extracted, CR: Composite Reliability

### Confirmatory factor analysis (CFA) sample

The CFA sample consisted of 496 nurses (n_CFA_=496), approximately 14 times the number of scale items. The factor loadings ranged from 0.690 to 0.890, indicating strong correlations with the underlying factor [56], and confirming the well-defined structure [57] (Table 3). The model fit indices indicated an acceptable fit: χ2/sd = 4.325, Root Mean Square Error of Approximation (RMSEA) = 0.078, Root Mean Square Residual (RMR) = 0.046, Turker Lewis Index (TLI) = 0.915, Comparative Fit Index (CFI) = 0.924, Normed Fit Index (NFI) = 0.903, Goodness of Fit Index (GFI) = 0.882, Incremental Fit Index (IFI) = 0.924 [55, 59, 60].

**Table 3:** The validity and reliability findings of the confirmatory factor analysis sample (N_CFA_=496)

Item No	Factor Loads	M	SD	Item Total *r*	α when item is removed	α	AVE	CR
NPTMS5- Nurses’ talent is determined using valid methods.	0.74	3.5081	1.04637	0.748	0.977			
NPTMS7- Talented nurses are provided with a work environment where they can demonstrate their talents.	0.80	3.4940	1.10826	0.802	0.977			
NPTMS8- Talented nurses are adequately compensated for their contribution to the organization.	0.72	2.9456	1.23871	0.732	0.978			
NPTMS9- Nurses are grouped according to their competencies and potential talents.	0.73	3.1633	1.10442	0.747	0.977			
NPTMS10- Talent management is an organizational policy.	0.70	3.4960	1.03669	0.706	0.978			
NPTMS11- Talented nurses are made to feel valued.	0.75	3.0827	1.23387	0.772	0.977			
NPTMS12- Training and development programs are organized according to the needs of the talented nurse.	0.75	3.3024	1.16743	0.757	0.977			
NPTMS13- The organization is known for its talented nurses.	0.69	3.3226	1.05255	0.686	0.978			
NPTMS15- Talented nurses with high adaptability to changing conditions are selected.	0.76	3.2923	1.08303	0.762	0.977			
NPTMS16- Nurses are given the opportunity to use their talents in their work.	0.79	3.4677	1.02640	0.781	0.977			
NPTMS18- Talented nurses are discovered through events for students and new graduates.	0.69	3.2036	1.13746	0.691	0.978			
NPTMS19- Nurses are given different roles and responsibilities in which they can develop their talents.	0.79	3.3387	1.08720	0.789	0.977			
NPTMS20- In recruitment, importance is given to matching the values of the organization with the individual values of talented nurses.	0.79	3.3448	1.03869	0.787	0.977	0.978	0.501	0.982
NPTMS21- Managers are aware of nurses’ talents.	0.79	3.3790	1.16232	0.792	0.977			
NPTMS22- Talented nurses are tried to be recruited to the organization.	0.84	3.3488	1.17779	0.841	0.977			
NPTMS23- Nurses’ talents are compatible with the position they work in.	0.81	3.3427	1.08594	0.796	0.977			
NPTMS24- Nurses are developed in a talent pool for future positions.	0.81	3.1996	1.11123	0.793	0.977			
NPTMS25- The talents needed for each nursing position are determined.	0.84	3.3367	1.04521	0.823	0.977			
NPTMS26- Emphasis is placed on the talents of nurses when assigning them in key positions.	0.83	3.3871	1.10616	0.808	0.977			
NPTMS27- There are development opportunities for career advancement for talented nurses.	0.86	3.3488	1.13588	0.843	0.977			
NPTMS29- Nurses who feel that they cannot use their talents sufficiently in their work are given the opportunity to work in a position that matches their talents.	0.80	3.1613	1.12913	0.821	0.977			
NPTMS30- The most talented nurses are selected to provide added value to the organization.	0.84	3.1794	1.12189	0.782	0.977			
NPTMS31-The achievements of talented nurses are recognized.	0.85	3.1734	1.18329	0.823	0.977			
NPTMS32- Nurses are assigned in areas/units suitable to their talents.	0.88	3.3105	1.07889	0.849	0.977			
NPTMS33- Nurses are given the opportunity to use different methods in their work to utilise their talents more effectively.	0.89	3.2540	1.07119	0.853	0.977			
NPTMS34- Managers contribute to the development of nurses’ talents.	0.89	3.3629	1.13589	0.862	0.977			

Note. NPTMS: Nurses’ Perception of Talent Management Scale, M: Mean, SD: Standard Deviation, r: Correlation, α: Cronbach’s alpha, AVE: Average Variance Extracted, CR: Composite Reliability

To assess the construct validity of the NPTMS in the EFA, the standardized factor loadings for each scale item and the exploratory factor (R²) associated with the items were examined. As expected, all items exhibited significant factor loadings (*p* < 0.05), with R² values ranging from 0.468 to 0.779. The standardized beta (β) coefficients for the items ranged from 0.684 to 0.883, indicating that the items adequately represented the exploratory factor and demonstrated high explanatory power [54, 61].

### Concurrent validity

In the EFA (*r* = 0.755, *p* < 0.05) and CFA (*r* = 0.772, *p* < 0.05) samples, the NPTMS exhibited a high positive correlation with the TMS scores [51].

### Convergent and divergent validity

In the EFA sample, the AVE was 0.619 (> 0.5) and CR was 0.987 (> 0.8), while in the CFA sample, AVE was 0.501 (> 0.5) and CR was 0.982 (> 0.8). These values confirmed that both convergent and divergent validity were achieved (CR > AVE) [52] (Tables 2 and 3).

### Reliability

The reliability of the NPTMS was assessed in EFA (n_EFA_=422) and CFA (n_CFA_=496) samples [36, 38] (Tables 2 and 3).

### Item-total score correlation

Item-total score correlations in both the EFA (0.690–0.863) and CFA (0.686–0.862) samples were > 0.60, indicating high correlations and confirming distinctiveness of the items [36, 37] (Tables 2 and 3).

### Split-half method

The results of the split-half method for the scale are presented in Table 4. A high level of reliability was found (> 0.70) [48, 49].

**Table 4:** Split-half analysis results of the scale (n_EFA_=422, n_CFA_=496)

		Cronbach’s α (the first half)	Cronbach’s α (the second half)	Guttman Split-Half	Spearman Brown
EFA sample		0.950	0.967	0.934	0.935
	Number of items	13	13	26	26
CFA sample	Number of items.	0.951 13	0.970 13	0.944 26	0.946 26

### Cronbach’s alpha

Cronbach’s α coefficients for the EFA (0.976) and CFA (0.978) samples were considered excellent (≥0.90) [38] and ideal (0.80–0.90) [48], respectively (Tables 2 and 3).

### Equivalent forms reliability

Equivalent forms reliability was demonstrated in the EFA (*r* = 0.755, *p* < 0.05) and CFA (*r* = 0.772, *p* < 0.05) samples, where the NPTMS showed a highly positive correlation with the TMS scores [51].

### Test-retest

The ICC coefficiant was 0.836, indicating good scale reliability (> 0.75–0.90) (*p* < 0.05) [62] (Table 5). A very high positive correlation was found between the retest scores of the NPTMS and the TMS (*r* = 0.868, *p* < 0.05) [51]. This suggests that the scale provides time-invariant measurements.

**Table 5:** Test-retest analysis results (*N* = 98)

	Group	*N*	M $\:\pm\:$SD	Median (Min-Max)	t	*p*	ICC (95%CI)/*p*
NPTMS	Test	98	116.3367$\:\pm\:$28.909	122.00 (45–170)	0.154	0.878	0.836 (-4.251/4.966) < *0.05
	Retest	98	115.9796$\:\pm\:$32.283	124.50 (34–170)			

Note. *p < 0.05, Paired Sample t-Test

NPTMS: Nurses’ Perception of Talent Management Scale, M: Mean, SD: Standard Deviation, Min: Minimum, Max: Maximum, ICC: Intraclass Correlation Coefficient, CI: Confidence Interval

### Final measurement scale

The NPTMS was finalized through comprehensive validity and reliability assessments. The scale consists of 26 items representing a single factor and was developed using a 5-point Likert-type scale [63]. Scores on the scale range from 1 (Strongly disagree) to 5 (Strongly agree), with higher scores indicating a higher level of perception of TM. The average scores were categorized as follows: “low” (1-2.346 points) “moderate” (2.35–3.653), and “high” (3.66-5). The final version of the scale is shown in Appendix 1.

### Nurses’ perception of talent management

The mean score of the CFA sample was 4.315 (SD 1.114, Min 1.73, Max 4.54), with a mode of 4.23 and a median of 4.692. Significant differences were found between private/foundation hospitals (4.496 ± 1.065), private/foundation university hospitals (4.646 ± 0.982), training and research hospital (3.903 ± 1.082) and public hospital (3.319 ± 0.992) (F = 2.876, *p* < 0.05). Analysis revealed significant differences between private/foundation hospitals and private/foundation university hospitals, as well as between training and research hospital and public hospital.

## Discussion

According to the Resource-Based View and Human Resources Architecture Approach, it is emphasized that talents that contribute to the organization’s value through the provision of qualified services play a key role in gaining a competitive advantage. These approaches highlight the necessity of attracting, developing, and retaining the best employees within organizations [12, 13]. In this context, given the rapid changes in healthcare systems, marked by increasing complexity, ambiguity, and uncertainty, there is an escalating need for a new and comprehensive approach to the identification, attraction, recruitment, placement, development, and retention of nursing talent that healthcare organizations may require in the future [2]. As a matter of fact, TM can serve as an effective tool for identifying, developing, and evaluating the talents of nurses [5]. SET, which fosters reciprocity between employees and organizations, also considers talent management as a significant investment made by an organization in its most valuable employees [39].

Existing TM measurement tools, developed for employees across various sectors (e.g., education, business), are insufficient for evaluating TM practices specifically in nursing. Therefore, there was a crucial need to develop a specialized, comprehensive, and practical measurement tool in the nursing context. In this regard, it was essential to clearly define and operationalize the concept of TM within the nursing context to develop this new scale. Through an extensive literature review, TM and its processes in nursing were defined from the broadest perspective. Although the scale was determined to be unidimensional, it comprehensively incorporates the essential practices of the TM process, including identification, attraction, recruitment, placement, development, and retention (e.g [10, 18, 20, 21]). Therefore, the items included in the NPTMS align with the Talent Factory Model [14] and Bersin’s New Talent Management Framework (2010) [15]. The scale allows for a comprehensive assessment of nurses’ perceptions of the TM process and facilitates an overall evaluation of TM practices based on the Classical Model: Systems Approach [16].

The construct validity of the NPTMS was tested separately using EFA and CFA on distinct samples. Similar to an existing scale [43], the single factor identified in this study was supported. The NPTMS, developed specifically within the context of nursing management and practices, considers the unique characteristics of nursing and offers a more comprehensive assessment of TM practices. The CFA results demonstrated acceptable fit indices, confirming the unidimensional structure of the scale [55, 59, 60]. GFI of less than 0.90 can be attributed to the increased number of items per factor [64]. Furthermore, the results of concurrent, convergent, and divergent validity analyses revealed the construct validity of the scale [51, 52]. To assess the reliability of the NPTMS, item-total score correlations, the split-half method, Cronbach’s α coefficient, equivalent forms reliability and test-retest reliability (two weeks interval) were examined across both the EFA and CFA samples. These analyses confirmed the reliability of the scale [36, 37, 48, 49, 51, 62]. In addition, a very high positive correlation between the NPTMS and TMS total scores suggested that the scale provides time-invariant measurements [51]. Based on the psychometric evaluation results, it is concluded that the NPTMS is a valid and reliable tool.

The scale has strengths for practical application, particularly in nursing services. In the development process of the scale, its feasibility for effective use in nursing services was attempted to be achieved through providing a simple, clear and understandable structure. The items were created based on a comprehensive review of the literature using the deductive method, ensuring that the scale includes the most comprehensive practices of the TM process, particularly in the context of nursing services. One of the key strengths of the scale is its ability to provide a holistic and organizational understanding of the TM process. Through examining TM from this broader perspective, the scale allows for a deeper inside into how TM practices impact nursing. This newly developed scale was designed to be applicable to all nurses and it can serve as a valuable tool for manager, nurse leader, and policymakers. It provides crucial data on nurses’ perceptions of TM, which can help evaluate the effectiveness of current TM practices and guide the development of new practices based on these insights.

The mean NPTMS scores obtained from the CFA sample were found to be high, similar to studies in Indonesia [65] and Iran [31] in the nurse sample. In this study, nurses working in private/foundation university hospitals obtained the highest average scores, while those working in public hospital had the lowest scores. In Egypt, nurses expressed satisfaction with TM practices, with hospitals successfully attracting, developing, motivating, and retaining talented employees, which contributed to a positive perception of TM [27, 30]. Arıcı [28] reports in a study consisted mostly of nurses in private hospital that nurses had high perceptions of being assigned to roles that match their talents. In contrast, a study in Poland reported that TM was not applied in health institutions [29]. In comparison to the current study, previous research has shown that nurses’ perception of TM is low [32, 34, 66] and above average [33].

The high level of perception of TM found in this study, compared to previous studies [32–34, 66], suggests that nurses in this sample have positive perceptions of TM in their institutions and consider that their talents are being recognized and assessed. This may also be due to the higher number of nurses working in private hospitals in the sample, as well as the fact that a significant number of nurses had less than one year of professional experience. Furthermore, the nursing-specific focus of the scale used in this study could be another contributing factor. Although there are no direct, formalized TM practices for nursing in Türkiye, several practices aimed at ensuring the professional development of nurses within their current roles are implemented across both public and private organizations. These include orientation training, increasing professional knowledge and skills, training activities for individual development programs, participation in conferences and congresses, courses and graduate education opportunities for special nursing fields [28], creating positive work environments and supporting individual career planning. In a study consisted mostly of nurse managers working in the public sector, it was determined that the most commonly used technique in career development was training programs. Promotion decisions for nurses were based on fundamental criteria such as expertise, talent and performance, educational level, and field of practice [23]. In addition, nurses working in public institutions are provided with a number of financial opportunities, as well as career advancement prospects, albeit limited, under the framework of the Civil Servants Law No. 657 [67], which applies to all public employees. Nurses who complete postgraduate education in their specialized field can achieve the title of specialist nurse, as per the updated Nursing Law of 2007 [68]. In these efforts to evaluate the nursing workforce, it is observed that the evaluation activities often lack a clear identification and distinction of nurses’ talents. It can also be concluded that TM practices in nursing are not approached as a holistic process.

### Limitations

There are several limitations to this study. First, the NPTMS, which is based on self-reporting by nurses, was applied in hospitals located in a single metropolitan province. This could introduce potential bias in the responses, since it reflect only the perceptions of nurses within a specific geographic era. In order to reduce this limitation, the research was conducted across a range of hospitals including public, private and university hospitals to ensure diversity in terms of professional human resources practices. Another limitation is that the cross-sectional data collection method, based on non-probability convenience sampling from 12 hospitals, may restrict the generalizability of the findings. Due to high service density of hospitals, nurses faced difficulties in allocating time to complete the data collection tools. Therefore, differences were observed between some demographic characteristics of the EFA and CFA samples. To reduce this limitation, the researcher made multiple visits to the hospitals in an attempt to reach all nurses during the data collection process. In addition, the higher the number of nurses working in private hospitals and those with less than a year of professional experience compared to others may have influenced the results. Nurses who had completed their trial and orientation period and had just started their work were likely more engaged in the development opportunities in the institution, which could have also contributed to their increased participation. In future studies, the scale can be used to assess TM practices in institutions and examine its impact on patient, nurse and organizational outcomes. It can be adapted to different languages and cultures for cross-national and international comparisons. It can serve as a tool in research exploring nurses’ perceptions of TM as an antecedent, mediator, or outcome variable.

## Conclusions

This study is the first to demonsrate that the 26-item, one-factor NPTMS, which comprehensively measures various practices involved in the TM process, is a valid and reliable tool to evaluate nurses’ perceptions of TM. In this regard, it fills the existing gap in the availability of measurement tools specifically designed to assess TM in nursing. The NPTMS will be valuable for evaluating TM practices in nursing services, supporting the development of human resource practices with a talent-focused approach, and informing policies and strategies that prioritize investments in nursing talent. It also provides researchers with an opportunity to compare nurses’ perceptions of TM on a global scale.

The results of this study have important practical implications for hospital managers, nursing leaders, and policymakers. Managers play a critical role in creating and sustaining a talent-oriented understanding/culture within their organizations. The NPTMS can provide valuable insights for hospital and nursing managers by helping them assess the extent to which TM practices are being implemented in nursing, identify strengths and weaknesses of current practices, and inform the development or refinement of TM strategies. The scale, which is applicable to all nurses, can also be used to evaluate nurse managers’ perceptions of TM at various organizational levels. Additionally, the NPTMS can offer policymakers data to support the development of policies and strategies aimed at investing in nursing talent and advancing the nursing profession.

## Electronic supplementary material

Below is the link to the electronic supplementary material.

Supplementary Material 1

Supplementary Material 2

## Data Availability

All data generated or analysed during this study are included in this published article. Data are available upon reasonable request from the first author.

## Abbreviations

AVE: Average Variation Extracted
CFA: Confirmatory Factor Analysis
CFI: Comparative Fit Index
CR: Composite Reliability
CVI: Content Validity Index
EFA: Exploratory Factor Analysis
GFI: Goodness of Fit Index
ICC: Intraclass Correlation Coefficient
ICN: International Council of Nurses
IFI: Incremental Fit Index
KMO: Kaiser–Meyer–Olkin
NFI: Normed Fit Index
NPTMS: Nurses’ Perception of Talent Management Scale
RMR: Root Mean Square Residual
RMSEA: Root Mean Square Error of Approximation
SET: Social Exchange Theory
TLI: Turker Lewis Index
TM: Talent Management
USA: United States of America
WHO: World Health Organization
