# Supplementary material for: Nurses’ perception of talent management scale (NPTMS): development, validation and psychometric properties
Source: BMC Nurs. 2025 Apr 3;24:366. doi: 10.1186/s12912-025-02882-8 (PMC11966793; doi:10.1186/s12912-025-02882-8)
Supplement: Supplementary file 1 — Supplementary Material 1 [file 12912_2025_2882_MOESM1_ESM.pdf]

Appendix 1. Nurses' Perception of Talent Management Scale

Nurses' Perception of Talent Management Scale

Answer the following items by evaluating **the practices in the organization where you are currently working.**		Strongly Disagree	Disagree	Neither Agree or Disagree	Agree	Strongly Agree
1.	Nurses' talent is determined using valid methods.					
2.	Talented nurses are provided with a work environment where they can demonstrate their talents					
3.	Talented nurses are adequately compensated for their contribution to the organization.					
4.	Nurses are grouped according to their competencies and potential talents.					
5.	Talent management is an organizational policy					
6.	Talented nurses are made to feel valued					
7.	Training and development programs are organized according to the needs of the talented nurse.					
8.	The organization is known for its talented nurses.					
9.	Talented nurses with high adaptability to changing conditions are selected.					
10	Nurses are given the opportunity to use their talents in their work.					
11	Talented nurses are discovered through events for students and new graduates.					
12	Nurses are given different roles and responsibilities in which they can develop their talents.					
13	In recruitment, importance is given to matching the values of the organization with the individual values of talented nurses.					
14	Managers are aware of nurses' talents.					
15	Talented nurses are tried to be recruited to the organization.					

		Strongly Disagree	Disagree	Neither Agree or Disagree	Agree	Strongly Agree
16	Nurses' talents are compatible with the position they work in.					
17	Nurses are developed in a talent pool for future positions.					
18	The talents needed for each nursing position are determined.					
19	Emphasis is placed on the talents of nurses when assigning them in key positions.					
20	There are development opportunities for career advancement for talented nurses.					
21	Nurses who feel that they cannot use their talents sufficiently in their work are given the opportunity to work in a position that matches their talents.					
22	The most talented nurses are selected to provide added value to the organization.					
23	The achievements of talented nurses are recognized.					
24	Nurses are assigned in areas/units suitable to their talents.					
25	Nurses are given the opportunity to use different methods in their work to utilise their talents more effectively.					
26	Managers contribute to the development of nurses' talents.					

Copyright © 2024 [Not presented for review] All rights reserved. Please do not use or copy this scale without written permission from the authors.
